# Main and Accessory Canal Filling Quality of a Premixed Calcium Silicate Endodontic Sealer According to Different Obturation Techniques

**DOI:** 10.3390/ma13194389

**Published:** 2020-10-01

**Authors:** Su-Yeon Ko, Hae Won Choi, E-Deun Jeong, Vinicius Rosa, Yun-Chan Hwang, Mi-Kyung Yu, Kyung-San Min

**Affiliations:** 1 Department of Conservative Dentistry, School of Dentistry and Institute of Oral Bioscience, Jeonbuk National University, Jeonju 54896, Korea; tastynice87@gmail.com (S.-Y.K.); jed0709@nate.com (E.-D.J.); mkyou102@jbnu.ac.kr (M.-K.Y.); 2 Department of Dental Biomaterials Science, School of Dentistry, Seoul National University, Seoul 03080, Korea; orthochoi7@gmail.com; 3 Department of Orthodontics, The Institute of Oral Health Science, Samsung Medical Center, Sungkyunkwan University School of Medicine, Seoul 06351, Korea; 4 Discipline of Oral Sciences, Faculty of Dentistry, National University of Singapore, Singapore 119085, Singapore; denvr@nus.edu.sg; 5 Department of Conservative Dentistry, School of Dentistry, Chonnam National University, Gwangju 61186, Korea; ychwang@chonnam.ac.kr; 6 Research Institute of Clinical Medicine of Jeonbuk National University, Jeonju 54907, Korea; 7 Biomedical Research Institute of Jeonbuk National University Hospital, Jeonju 54907, Korea

**Keywords:** accessory, canal, calcium silicate, endodontic, sealer, ultrasonic

## Abstract

The present study aimed to investigate the effects of different obturation techniques on the main and accessory canal filling quality of a premixed calcium silicate endodontic sealer (Endoseal TCS). We also highlighted the validity of the methods used for evaluating the canal filling quality. Thirty single-rooted premolars were used for the main canal filling and 75 were used for accessory canal filling. The canals were instrumented and randomly divided into three groups according to the filling techniques: (1) single-cone technique (SC), (2) single-cone with ultrasonic activation (SU), and (3) warm vertical compaction (WV). Voids in relation to the root canal fillings were assessed using cross-section images from microcomputed tomography (μCT) scans or transversely sectioned tooth specimens (*n* = 10). After demineralization and clearing of the teeth, the incidence, number, and completeness of the accessory canal fillings were evaluated (*n* = 25). One-way analysis of variance (ANOVA) and Tukey’s post hoc test was used for the evaluation of the voids in the main root canal and the incidence and number of filled accessory canals. Pearson’s chi-squared (χ^2^) test was used for the evaluation of the filling completeness (α = 0.05). In the stereomicroscopic evaluation of the sectioned specimen, the SC group had significantly higher void occurrence than the other groups (*p* < 0.05), although there was no difference between groups in the μCT evaluation. However, there was no difference between the SU and WV. There was no difference between all the groups regarding the incidence, number, and completeness of the accessory canal fillings. When the premixed calcium silicate sealer is used with SC, the ultrasonic activation is recommended to obtain a better main canal filling quality. In contrast, the obturation techniques did not affect the accessory canal filling. We also recommend using the sectioning method when the void formation in the root canal filling materials is evaluated.

## 1. Introduction

A complete fluid-tight seal of the root canal system determines an endodontic treatment’s long-term success after a biomechanical preparation by preventing oral pathogens from reinfecting the root canal and periapical tissues. Therefore, clinicians should choose an obturation technique that will provide a three-dimensional seal of the entire root canal. In addition to the main root canals, accessory canals also create potential pathways through which bacteria can spread and remain unaffected by treatment procedures [1]. Therefore, it is advocated that a canal filling should be up to the last few millimeters of the apical area and accessory canals.

Conventionally, a core material, generally gutta-percha (GP), is used along with a root canal sealer that seals the core and dentin interface. Several types of endodontic sealers are currently available in the endodontic market. Among these, calcium silicate sealers are of great interest because of their favorable biocompatibility [2] and antimicrobial effects [3]. Furthermore, the setting of a calcium silicate sealer is less affected by the moisture than other types of sealers, where maintaining moisture in the root canal may be advantageous before filling the root canals [4].

For long periods, many variations of lateral compaction and warm vertical compaction techniques have been widely used for the obturation of root canals. However, these techniques might be time-consuming and require complicated equipment. Moreover, the force resulting from the compaction can damage the root dentin [5]. Meanwhile, the single-cone (SC) technique has emerged as a simple alternative, mainly when calcium silicate sealer is used. In the SC technique, the GP cone acts just as a carrier and path for retreatment. Furthermore, the SC technique is less operator-dependent and potentially reduces the damage to dentinal walls. According to one survey, among calcium silicate sealer users, the SC technique was the most employed obturation method applied by general dental practitioners [6].

Despite being easier to use and requiring less time spent on obturation, according to our previous study, the SC technique may leave several voids in irregularly shaped canals [7]. These voids are of great concern as they reduce the filling quality and can serve as hubs for microbial housing, leading to reinfection and treatment failure [8]. In this respect, we previously introduced a sophisticated method using an ultrasonic-mediated SC technique to reduce void formation [7]. We showed that the technique was more beneficial to obturate the root canal with less void formation compared to warm vertical compaction. However, in contrast to our study, a considerable number of recent studies have reported that the filling quality of the SC technique was not inferior but instead superior to other techniques, such as warm vertical compaction [9,10,11,12,13,14,15,16]. While reviewing the papers, we recognized that all the studies were performed only using microcomputed tomography (μCT) but did not observe the sectioned tooth specimens using a microscope. Notably, in our previous study, we suggested that traditional microscopic observation with a suitable scoring system was more reliable than μCT analysis [7]. In this respect, we felt the necessity to re-evaluate the SC technique’s filling quality compared to the ultrasonic-mediated or warm vertical compaction technique. Furthermore, in the present study, we evaluated the quality of accessory canal obturation to provide additional information regarding the effect of the obturation techniques on the microanatomical structure since there has been no related study.

Therefore, this study aimed to investigate the effect of the aforementioned obturation techniques on the filling quality of a calcium silicate sealer (Endoseal TCS; Maruchi, Wonju, Korea), which was recently developed as a white version of the previous grey-colored product (Endoseal MTA; Maruchi) using both μCT and stereomicroscopic observations. We also evaluated the effect of the techniques on the accessory canal filling quality. 

## 2. Materials and Methods 

### 2.1. Preparation of theTeeth

A total of 105 extracted single-rooted teeth with a ribbon-shaped canal were obtained with patient informed consent under a protocol approved by the Institutional Review Board of Jeonbuk National University Hospital (No. 2019-06-058). Thirty teeth were assigned for the main canal obturation, and 75 teeth were used for accessory canal fillings. All teeth were decoronated at the cementoenamel junction and adjusted such that each root was approximately 15 mm in length. Subsequently, a size #10 K-File (Dentsply-Maillefer, Ballaigues, Switzerland) was inserted into the root canal until the tip was just visible beyond the apex. The working length was determined by subtracting 0.5 mm from this length. The teeth were then instrumented to a size F3 using ProTaper Universal NiTi rotary instruments (Dentsply-Maillefer). The root canals were irrigated with 5% sodium hypochlorite combined with passive ultrasonic irrigation (Endosonic blue, Maruchi) for 10 min and 1 mL of 17% ethylenediaminetetraacetic acid (EDTA; Wako Chemical, Osaka, Japan) for 1 min. Then, the canals were dried with paper points (Diadent, Cheongju, Korea).

### 2.2. Canal Filling Procedure

**Figure 1:**
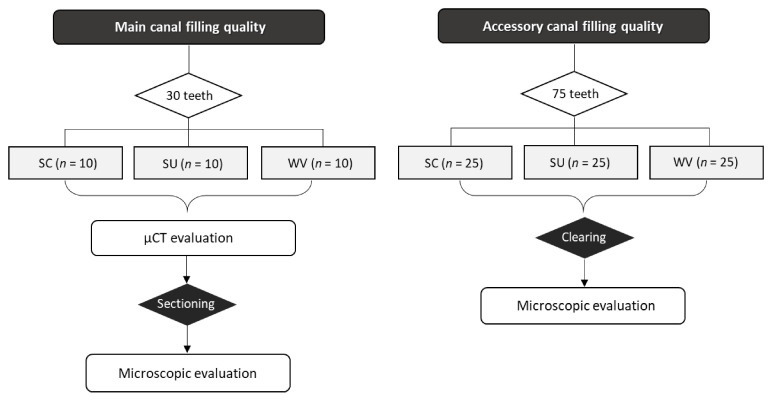
Flowchart representing the experimental procedure. SC: single-cone technique, SU: single-cone technique with ultrasonic activation, WV: warm vertical compaction.

**Figure 2:**
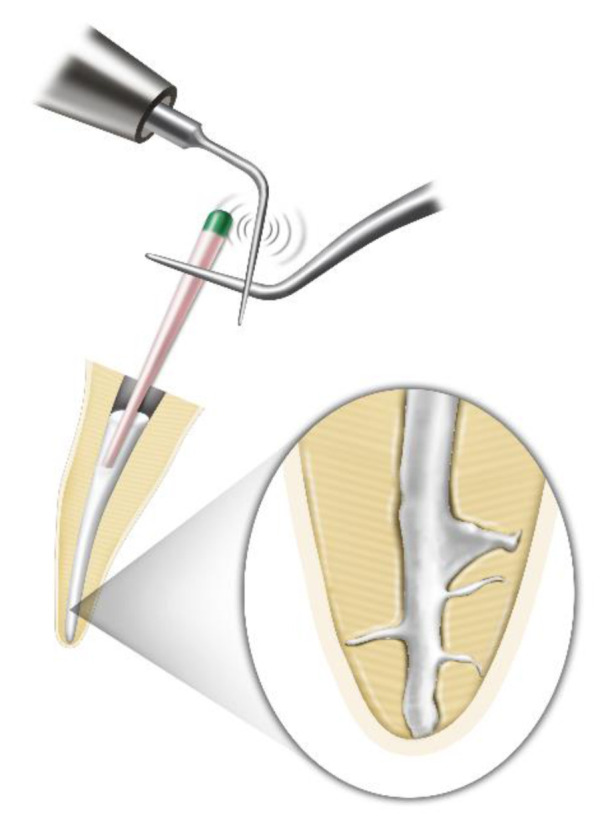
Illustration of the ultrasonic-mediated activation for the main and accessory canal fillings.

Teeth were assigned into three experimental groups according to the filling techniques (Figure 1):

- SC group (SC technique): We applied the sealer up to one-third of the root level from the apex and slowly inserted the selected GP cone (#35/.04) (Diadent) into the canal.
- SU group (SC technique with ultrasonic activation): We connected an ultrasonic tip (StartX #3, Dentsply-Maillefer) to an ultrasonic device (P-5 Newtron XS; Satelec, Mount Laurel, NJ, USA), which was set on “8” in the yellow code (i.e., indicated as suitable for endodontics by the manufacturer). Then, we applied ultrasonic vibration to a cotton plier that held the GP cone 20 mm from the tip after placing the sealer into the canal. Then, we inserted the GP cone slowly with ultrasonic activation. The ultrasonic application time was 3 s (Figure 2).
- WV group (warm vertical compaction): After each canal was filled with the sealer and GP cone, the inserted GP cone was compacted using SuperEndo B&L Alpha II (B&L Biotech, Ansan, Korea) to within 3 to 5 mm of the working length. We then backfilled the canal using SuperEndo B&L beta (B&L Biotech).

The access cavity was sealed with a composite resin (G-ænial Flo; GC, Tokyo, Japan), and the teeth were stored at 37 °C and 100% humidity for 7 days. 

### 2.3. μCT Analysis

The teeth were scanned using a high-resolution μCT scanner, SkyScan 1076 (SkyScan, Kontich, Belgium) at 100 kV and 100 μA, with a pixel size of 30 μm and a 0.5 mm aluminum filter. The scanning was performed via a 180° rotation around the vertical axis with a rotation step of 0.4° and an exposure time of 316 ms. Before each scan, the detector was air-calibrated to minimize ring artifacts. Beam-hardening corrections and contrast levels were adjusted in accordance with the manufacturer’s instructions. The axial cross-sections of the samples were reconstructed using NRecon (SkyScan) software. Each section was evaluated by two observers who were unaware of the filling techniques on a diagnostic screen to assess the presence of voids. The mean was calculated in each section and the proportion of voids was computed.

**Figure 3:**
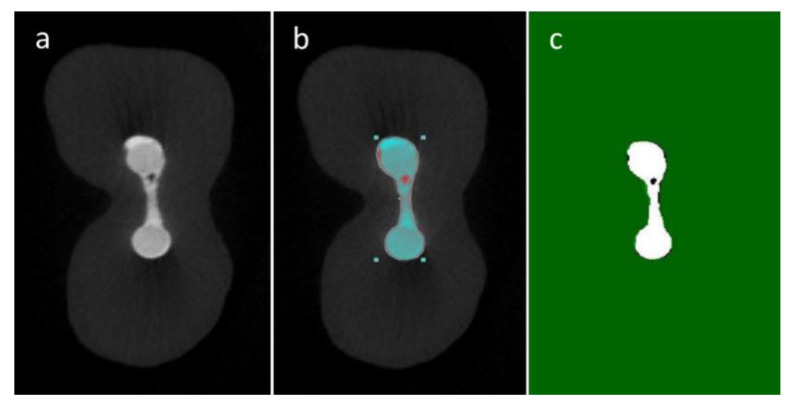
Representative microcomputed tomography (μCT) images showing (**a**) the marked defect, (**b**) the region of interest (ROI) selection on the images, and (**c**) the void detection inside the ROI.

To measure the volume of the voids, the original grayscale images were processed using a slight Gaussian low-pass filter for noise reduction and an automatic segmentation threshold was applied using CT-An version 1.12.9 (SkyScan) software. This thresholding process helped to select a range of gray levels for GP, dentin, voids, or sealer to obtain an image in black and white pixels. Then, the region of interest (ROI) was selected. The percentage of voids (V%) was calculated as V% = Vv/(Vv + VM) × 100 (Vv—volume of void, VM—volume of the filling material) (Figure 3).

### 2.4. Stereomicroscope Analysis

**Figure 4:**
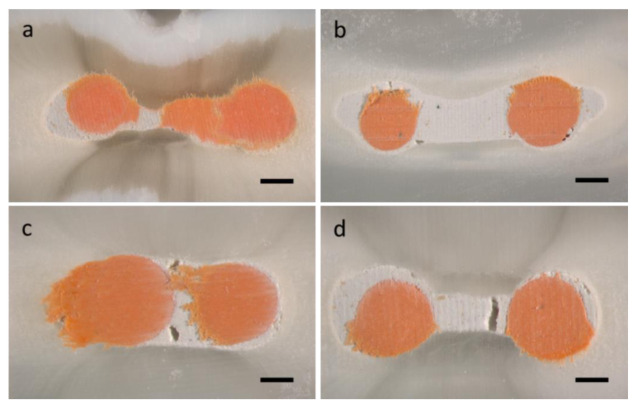
Representative images that were observed under a stereomicroscope: (**a**) score 1 from the WV group, (**b**) score 1 from the SU group, (**c**) score 2 from the SU group, (**d**) score 4 from the SC group. WV: warm vertical compaction, SU: single-cone technique with ultrasonic activation, SC: single-cone technique. Scale bar: 200 μm.

After the μCT examination, each sample was sectioned into six slices of 1.5 ± 0.1 mm each using a diamond-coated saw (Isomet; Buehler, Chicago, IL, USA). Then, every two slices were obtained from the coronal, middle, or apical parts. These slices were analyzed using a stereomicroscope (MZ16FA; Leica Microsystems, Wetzlar, Germany) and photographed. The number of voids was counted from these photographs and scored (Table 1). The scoring was evaluated by two observers who were not aware of the filling methods used (Figure 4). Measurements were done twice and the mean was calculated for each section.

### 2.5. Sample Clearing for the Evaluation of Accessory Canal Fillings

Seventy-five incisors with round canals were immersed in 7% nitric acid for 3 days for demineralization. The solution was changed every day and agitated occasionally. They were rinsed in running tap water for 4 h and immersed in 80% ethanol solution overnight, rinsed in distilled water, dehydrated in ascending concentrations of ethanol at 90% (1 h), 100% (1 h, three times each), and finally cleared and stored in methyl salicylate (Wako Chemical). 

### 2.6. Evaluation of the Incidence, Number, and Completeness of the Accessory Canal Fillings

**Figure 5:**
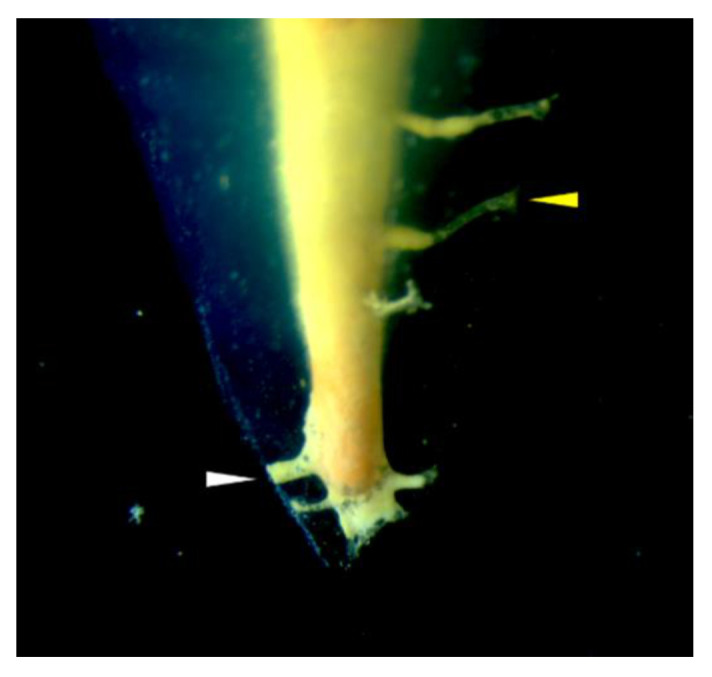
Representative image of a transparent root apex showing complete (white arrow) and partial (yellow arrow) filling of an accessory canal.

Analysis of four root surfaces of the apical 3 mm under a digital stereomicroscope (MZ16FA; Leica Microsystems) was conducted. The number of roots that showed accessory canal(s) among all the roots (incidence) was recorded. The number of observed accessory canals in each root was counted. Furthermore, the completeness of the accessory canal fillings (complete or partial) was evaluated. When the canal was filled with the sealer onto the external canal orifice, it was recorded as a complete filling, as indicated in Figure 5. Otherwise, it was considered as a partial filling.

### 2.7. Statistical Analysis

The sample size was determined using G-Power 3.1 software (University of Düsseldorf, Düsseldorf, Germany). A power analysis using the F test was applied, which identified the required sample sizes. All statistical analyses were performed using SPSS version 12 (SPSS Inc, Chicago, IL, USA). One-way analysis of variance (ANOVA) and Tukey’s post hoc test were used for the evaluation of the voids in the main root canal and the incidence and number of filled accessory canals. Pearson’s chi-square (χ^2^) test was used for the evaluation of the filling completeness. Differences were considered statistically significant at *p* < 0.05. Prior to the analyses, the Kolmogorov–Smirnov test was used for determining whether the data had a normal distribution.

## 3. Results

### 3.1. Evaluation of the Main Canal Filling Quality

#### 3.1.1. μCT Analysis

The mean percentage of μCT sections with void and volume percentages of the void are summarized in Table 2. Voids were found in all the groups and there was no significant difference between groups (*p* > 0.05).

#### 3.1.2. Stereomicroscope Analysis

In contrast to the findings from the μCT, significant differences were found between the groups regarding the average number of void and void scores. As shown in Table 3, the SC group had the highest number of voids and void scores (*p* < 0.05). However, there was no significant difference between the SU and WV groups in their number of voids and void scores (*p* > 0.05).

### 3.2. Evaluation of the Accessory Canal Filling Quality

#### 3.2.1. Incidence and Number of Accessory Canals

As shown in Table 4, accessory canals were found in 16 (64%) teeth using SC, 20 (80%) teeth using SU, and 14 (56%) teeth using WV, respectively. Furthermore, the average number of canals was 1.96 ± 2.37 using SC, 2.12 ± 2.08 using SU, and 1.60 ± 2.04 using WV, respectively. However, there was no statistical difference between the groups (*p* > 0.05).

#### 3.2.2. Completeness of the Fillings

The completeness of the accessory canal fillings (partial or complete) was recorded using the stereomicroscope images. Table 4 showed the percentage of accessory canal filling with the partial or complete filling rate for each group, where the highest percentage with the complete filling rate was for SC (65.3%), followed by WV (57.5%) and SU (52.1%). There was no significant difference between the groups (*p* > 0.05).

## 4. Discussion

Premixed calcium silicate sealers have gained the attention of clinicians because they can be used with the SC technique, which is easy to use and less time-consuming. According to some previous studies, the SC technique showed favorable bond strength and leakage resistance [17,18]. However, in terms of void formation, a couple of studies, including ours, reported that the SC technique might be inferior to other techniques, such as the core-carrier method or vertical compaction [7,19]. Nevertheless, a significant number of studies showed that the SC technique did not generate more voids compared to other methods, and thus there are still controversies [9,10,11,12,13,14,15,16]. Therefore, in the present study, we reproduced the experiment using a newly launched zirconium-oxide-containing premixed calcium silicate sealer (Endoseal TCS) with similar teeth and protocol. We also evaluated the accessory canal filling ability of the SC, SU, and WV techniques.

In the μCT assessment, as we expected, there were no significant differences in the mean void percentage between the three different techniques (*p* > 0.05). We have speculated that the sealers are radiopaque and they may affect the μCT detection of voids within the bulk of the root filling. Therefore, we do not consider this method as being suitable for the evaluation of the voids within the filling material, although it is known to be highly accurate, quantitative, and non-destructive [7]. Similar to our previous study, when the same samples were subjected to stereomicroscopic evaluation, the SC group showed a higher number and score of voids than the SU and WV groups (*p* < 0.05), whereas there were no significant differences in the number of voids between the SU and WV groups (*p* > 0.05). This might be because of the transmission of acoustic microstreaming energy to the sealer, reducing the surface friction between the sealer particles and improving the flow and quality [20]. This study emphasized the importance of microscopic observations for evaluating the filling quality of radiopaque root canal sealers. 

We found that the studies that suggested the SC technique as a favorable method only used μCT evaluation without microscopic observation of the sectioned specimens. Interestingly, we found a study that utilized the same material (Endoseal MTA with/without ultrasonic activation) and methodology (sectioning method and same scoring system) as our previous and present studies [21]. The authors reported the same results as our studies. They showed that the SC technique causes a higher number of voids compared to the warm vertical compaction technique but is as effective as the warm vertical compaction when used along with ultrasonic activation. Based on the reproducible results conducted by independent research groups, we recommend the application of ultrasonic energy to reduce void formation when the SC technique is used. Moreover, an international survey on the use of calcium-silicate-based sealers reported that endodontic specialists utilized more of the thermoplasticized obturation techniques than the SC technique [6].

In the current study, we also investigated the effects of the obturation methods on the accessory canal filling quality. Unlike the main canal filling quality, the incidence and number of accessory canals were similar, regardless of the techniques used (*p* > 0.05). These results mean that the penetration of the sealers depends mainly on the hydraulic force applied by the insertion of the GP cone. In other words, if the orifice of the accessory canal is opened, the sealer can be inserted into the minute anatomical space. Furthermore, we found that the vertically applied compaction force did not increase the completeness of the filling (*p* > 0.05). This result also implies that the accessory canal filling process occurred at the first attempt and did not proceed with additional compaction. In this respect, the removal of remnants that blocked the orifice of the accessory canal by irrigation was much more important than the force applied by the filling instruments, such as pluggers. The use of EDTA and passive ultrasonic irrigation might also be crucial for the filling because it can remove the smear layer and debris, which cover the accessory canal orifice. 

We used the clearing technique to observe the filled accessory canals. This method has been employed to obtain information on root canal morphologies and is considered to be suitable for the observation of the accessory canal since it provides a very clear visualization of the canal anatomy, which was evident in the photographs taken [22,23,24]. Therefore, we could evaluate not only the number of canals but also the completeness of the filling in detail. One possible problem caused by this technique, especially in this study, might be the loss of calcium-silicate-based material during the demineralization procedure. The nitric acid can remove the mineral component from the material at the accessory canal orifice and might affect the evaluation of the completeness of the filling. However, according to the manufacturer’s information, the filling contains more than 50 wt% zirconium oxide to provide enough radiopacity and avoid tooth discoloration [25]. This white material is a kind of heavy metal and is not removed by the demineralizing agent. Therefore, we could claim that the result was not affected by the clearing technique. 

## 5. Conclusions

We suggest the application of ultrasonic energy when the SC technique is used with calcium silicate sealers. Furthermore, we recommend that researchers use the sectioning method when the void formation in the root canal filling materials is evaluated. However, the SC technique was comparable to the ultrasonic-mediated technique and warm vertical compaction in terms of the accessory canal obturation. Therefore, we conclude that the SC technique has the potential to be used as a useful method for root canal filling if it is adequately applied. Furthermore, further studies are required to evaluate the adhesion of the sealer to dentin or GP.

## Figures and Tables

**Table 1:** Scoring system for the evaluation of the filling quality.

Score	Characterization
1	Well-condensed filling shows only a few small voids (less than 0.1 mm in diameter)
2	Imperfectly condensed filling showing some small voids (more than 3) or middle-sized bubbles (0.1 mm to 0.2 mm in diameter)
3	Inadequately condensed filling showing many small voids (more than 5) or large bubbles (more than 0.2 mm in diameter)
4	Poorly condensed filling showing many small voids (more than 7) or empty space connecting separate canal walls.

**Table 2:** Mean percentage and standard deviation (SD) of sections with voids in two-dimensional slices and the volume percentage of the voids in three-dimensional images.

Criteria	Technique	Mean ± SD
Proportion of the section with voids (%)	SC	73.33 ± 16.1
	SU	69.99 ± 17.2
	WV	58.33 ± 22.5
Root filling (%)	SC	94.45 ± 5.13
	SU	95.09 ± 3.94
	WV	95.53 ± 3.78

There was no significant difference between the groups (*p* > 0.05). SC: single-cone technique, SU: single-cone with ultrasonic activation, WV: warm vertical compaction.

**Table 3:** The number of voids and scoring results of the voids in the sectioned specimens (mean ± SD).

Group	Number of Voids	Score of Void
SC	2.60 ± 1.99 ^A^	1.98 ± 1.06 ^a^
SU	1.80 ± 1.58 ^B^	1.58 ± 0.80 ^b^
WV	1.28 ± 1.32 ^B^	1.42 ± 0.74 ^b^

Groups identified by the same letter were not significantly different (*p* > 0.05). SC: single-cone technique, SU: single-cone with ultrasonic activation, WV: warm vertical compaction.

**Table 4:** Incidence, number, and completeness of the accessory canal fillings.

Category	SC	SU	WV
Incidence	16/25 (64%)	20/25 (80%)	14/25 (56%)
Number	1.96 ± 2.37	2.12 ± 2.08	1.60 ± 2.04
Completeness (%)	65.3	52.1	57.5

There was no statistical difference (*p* > 0.05). SC: single-cone technique, SU: single-cone with ultrasonic activation, WV: warm vertical compaction.
